# Selectivity of weak intermolecular forces and precursor state of elementary oxidation reactions, a new insight on Ne^*^ + N_2_ chemiionization

**DOI:** 10.1038/s41598-021-98602-8

**Published:** 2021-09-27

**Authors:** Stefano Falcinelli, Franco Vecchiocattivi, Fernando Pirani

**Affiliations:** 1 Department of Civil and Environmental Engineering, University of Perugia, Via G. Duranti 93, 06125 Perugia, Italy; 2 Department of Chemistry, Biology and Biotechnologies, University of Perugia, Via Elce di Sotto 8, 06123 Perugia, Italy

**Keywords:** Physical chemistry, Chemical physics, Reaction kinetics and dynamics

## Abstract

This paper reports on the collision dynamics of N_2_ with metastable Ne^*^ promoting chemiionizations, prototype of barrier-less oxidation reactions of great interest for fundamental and applied research. Extending guidelines presented in previous papers for the atom–atom case, an innovative treatment of the reaction stereodynamics involving molecules in a quantum state-to-state resolution conditions is proposed that emphasizes the role of structure and stability of the precursor that is here the reaction transition state. A critical test of such treatment, carried out exploiting a new formulation both of real and imaginary parts of the optical potential driving the reaction dynamics, is represented by the detailed-combined description of all relevant findings, provided by high resolution molecular beam scattering experiments carried out in our and other laboratories. The present analysis casts light on basic electronic rearrangements of such prototype oxidation reaction which are expected to be of fundamental interest for many other reactions involving open shell atoms and free radicals.

## Introduction

Weak chemical and physical intermolecular forces generate the precursor state of chemical-physical phenomena. In myriad of situations the precursor state of elementary gas-phase processes corresponds to a weakly bound adduct formed by collisions of interacting partners. Often, structure and stability of the precursor state are not available at the level of detail required to control the subsequent selective evolution towards the final states of the processes. This state defines a sort of electromagnetic “trap” by intermolecular forces, where the quantum confinement of the system determines outer electrons rearrangements and changes in their angular momentum couplings. The interest of modern research in Chemistry on such topic is emerging, especially when focused on low temperature processes (cold Chemistry) strongly affected by quantum effects^1–5^.

Chemiionization reactions (CHEMI), also known as Penning and autoionizations processes, are triggered by collisions of A^*^ species, electronically excited in highly energetic metastable states (usually He^*^ and Ne^*^ atoms which are prototype of open-shell species), with other atoms/molecules M^6,7^. In most cases, they form Penning ions M^+^ and/or associate ions AM^+^ plus electrons even if ionization rearrangements and fragmentation of M^+^ can occur in CHEMI involving molecules^7^. The precursor state of CHEMI directly controls the transition state formation of prototype single step barrier-less exothermic oxidation reactions of great interest for fundamental and applied research. Indeed, CHEMI participate to the balance of phenomena occurring in interstellar environments, in combustion and flames^8^, in molecular plasmas and nuclear fusion. They also govern the chemistry in space and planetary ionospheres^9,10^ affecting the transmission of radio and satellite signals. Particular attention has recently addressed to CHEMI occurring under ultra-cold conditions, investigated in detail with the merged molecular beams technique, in order to determine the nature of quantum resonances accompanying reactive and non-reactive collisions^1–5,11,12^.

As nuclear reactions^13^, CHEMI are driven by an optical potential that promotes both the precursor state formation and the following disappearance of reagents when they pass to product channels^6,7^. For most of CHEMI, detailed information on the optical potential, including full and internally consistent formulation of its real and imaginary parts, is still lacking.

Recently, a new theoretical approach, proposed by us and applied to the atom–atom Ne^*^(^3^P_2,0_) + Ar, Kr and Xe reactions^14–16^, pointed out that such CHEMI occur via two different microscopic mechanisms, whose relative importance depends on the quantum state of reagents and products and is modulated by kinetic energy *E* available for reagents and/or by interatomic (separation) distance *R* mainly probed during the collision events. The proposed approach, exploiting the interdependence of both real and imaginary part of the optical potential on the leading components of the intermolecular forces involved, casted light on important stereodynamics effects, like the role played by the atomic orbital alignment of reagents and products during the reaction evolution. Elementary processes have been also described in terms of confinement and/or transition between Hund’s cases of rotating diatoms, formed by the interacting systems during each collision event^17^, and the selectivity of all state-to-state reaction channels has been properly identified^16^.

This paper refers on a basic generalization step of such approach to the important case of atom-molecule CHEMI. In particular, the focus is on the prototype Ne^*^(^3^P_2,0_) + N_2_ reaction, for which many experimental information, obtained in the last 35 years in our and other laboratories with different apparatuses exploiting the molecular beam (MB) technique, is available^18–24^. Here, we give for the first time an internally consistent rationalization of all experimental findings, providing basic features of the optical potential that include its dependence on atomic alignment and molecular orientation with their selective role on the stereodynamics of each state-to-state reaction channel. Basic details on the electronic rearrangements triggering the reaction are also obtained, considered of crucial interest for many other reactions involving open shell species and occurring from sub-thermal up to hyper-thermal conditions.

## Methods: The optical potential

CHEMI are then driven by an anisotropic optical potential *W*^6,7,25,26^, defined in Eq. (1) as combination of a real (*V*_*t*_) and an imaginary (*Γ*) part:

1 $$ W = V_{t} - \frac{i}{2}\varGamma $$

While *V*_*t*_ essentially controls the collision dynamics in entrance channels, *Γ* mediates the passage from neutral reactants to ionic products through electronic rearrangements within the reaction transition state (TS). Therefore, *Γ* accounts for the “opacity” of the system. The strength of both *V*_*t*_ and *Γ* components varies with the center-of-mass separation *R* and the relative orientation of interacting partners.

**Figure 1:**
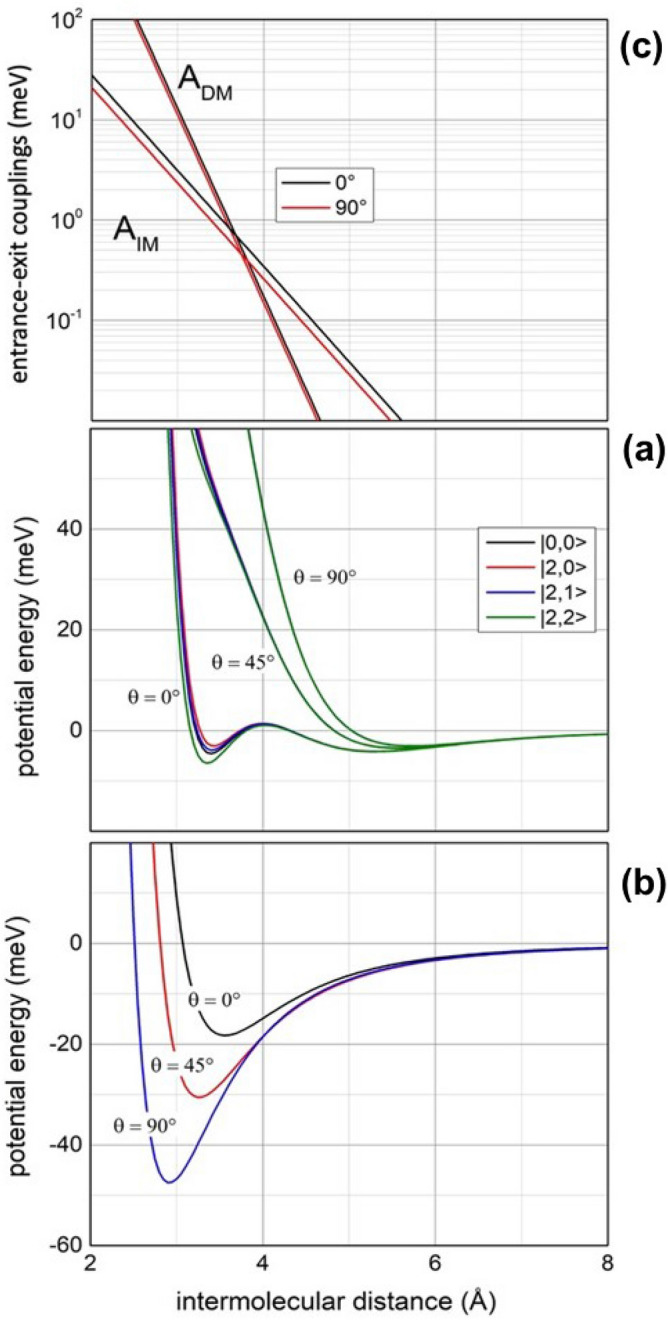
(**a**) Radial and angular dependence of the real part ${V}_{t}$ in each entrance channel. (**b**) The potential behaviour in the exit channel. (**c**) Radial and angular dependences of *A*_*DM*_ and *A*_*IM*_ couplings. The representation of the interaction potential for entrance and exit channels and details on the formulation of the various components is provided in (SI): it has been obtained extending that recently proposed for atom–atom reactions^15,16^.

We define ${V}_{t}$ in the entrance channels as the combination of a neutral–neutral dispersion attraction (dominant at large *R*) with size repulsion, attenuated by external electron cloud polarization that discloses to internal ionic core of metastable atom (such effect is emerging at intermediate-short *R*)^15,16^. We also enclose spin–orbit couplings (SO), charge transfer (CT) (due to configuration interaction between Ne^+^–N_2_ and Ne–N_2_^+^states), and atomic ion-molecular quadrupole electrostatic effects. In the exit channels the interaction is defined as that of a canonical neutral atom-molecular ion system, determined as balance of size repulsion with dispersion-induction attraction, perturbed by CT. Most of the involved interaction components depend also on the mutual orientation of the involved collision partners and therefore they are strongly anisotropic. Details on the formulation of ${V}_{t}$ are given in the Supplementary Information (SI), while the obtained dependence on *R*, on the molecular orientation (defined by the *θ* angle between the molecular axis (bond) orientation and **R**) and on the $|J,\varOmega >$ quantum states of the open shell Ne^*^(^3^P_J_) reagent is plotted in panel a of Fig. 1. As usual, *J* is the total (orbital + spin) electronic angular momentum quantum number, while Ω defines the absolute projection of **J** along **R**). The interaction in the exit channels, coupled by CT with the entrance channels, is obtained as an extension (see SI) of the formulation discussed in detailed for the near resonant Ar^+^-N_2_ ↔ Ar-N_2_^+^ system^27^. Results obtained for Ne-N_2_^+^, evaluated at specific molecular orientations, are plotted in panel b of the same Fig. 1.

The extension of the atom–atom treatment^14–16^ to the atom-molecule CHEMI suggests that the most effective electron rearrangements, triggering the reactivity through *Г*, are determined by the *A*_*DM*_ and *A*_*IM*_ quantities. They represent entrance-exit channel couplings promoting, respectively, the *direct mechanism* (*DM*), where the chemical reactivity (oxidation) is triggered by an effective electron exchange between reagents, promoted by CT, and the *indirect mechanism (IM*), stimulated by interaction contributions of physical nature^14–16^, as external electron cloud polarization, SO sublevels perturbation, with their mixing-decoupling stimulating photoionization, and Coriolis couplings*.* The latter relate to the centrifugal contributions that accompany any scattering event. Starting from the formulation of the imaginary part of atom–atom reactions^15,16^, the following relations are obtained for state-to-state *Γ* components controlling present CHEMI:

2 $$ \begin{aligned} \varGamma_{|0,0 \to ions > } & = A_{DM} C_{x} + + A_{IM} \left( {1 - C_{x} } \right) \\ \varGamma_{|2,0 \to ions > } & = { }A_{DM} \left( {1 - C_{x} } \right) + { }A_{IM} { }C_{x} \\ \varGamma_{|2,1 \to ions > } & = { }A_{DM} { }\frac{3}{4}{ }\left( {1 - C_{x} } \right) + { }A_{IM} { }\left( {\frac{3}{{4{ }}}C_{x} + \frac{1}{4}} \right) \\ \varGamma_{|2,2 \to ions > } & = { }A_{IM} { } \\ \end{aligned} $$

The coefficient ${C}_{x}$ is considered as a proper *marker-tracing* of the system evolution along each reaction channel^16^ whose value, radial and angular dependences are directly obtainable from the interaction potential formulation (see Supplementary Information, SI). More in detail, *C*_*x*_ accounts for the change of the angular momentum coupling of the open shell atom within the intermolecular electric field, whose strength and anisotropy varies with *R* and with the mutual orientation of both reagents. As in the atom–atom case^15,16^, where it also accounts for the system confinement in specific Hund’s cases^17^, ${C}_{x}$ represents the alignment degree of the half-filled orbital of the open shell Ne^+^ ionic core within the collision complex. In particular, ${C}_{x}=1$ *and* ${C}_{x}=0$ define respectively states of ^3^Σ and ^3^Π symmetries in the C_∞v_ (linear) configuration of the system, that splits in ^3^A_1_, ^3^B_1_ and ^3^B_2_ symmetries in the C_2v_ (perpendicular) configuration. Note that Σ and Π symmetries are defined by the molecular quantum number Λ = 0 and Λ = 1, respectively. In turn, such symmetries correlate with states ^3^A′, ^3^A′ and ^3^A″ of the more general C_s_ configuration.

As for atom–atom cases, the ${C}_{x}$ value depends on the competition between anisotropic CT and SO interaction contributions (see details in SI). For $0<{C}_{x}<1$, above mentioned symmetries are mixed by the SO coupling which determines the atomic sublevels formation and perturbation. For further details see Fig. 3 of ref.^15^ and Fig. 2 of ref.^16^. After all, such *marker-tracing* coefficient is the equivalent of the *indicator* in acid–base reactions: as the indicator provides information on the prevalence of acid or basic character and on the equivalence point, the present *marker-tracing* suggests if the precursor state is confined in that of weakly interacting complex, in that of a formed molecule or in the transition between the two limiting structures.

Obtained *A*_*DM*_ and *A*_*IM*_ coupling terms, plotted in Fig. 1c, are represented by exponential functions with completely different exponent since associated to interaction components of different nature (see above and SI). As we have extensively presented in detail in our general treatment for stereo-dynamics of state-to-state CHEMI^15^, it is important to point out that overlap effects with the continuum wave function of emitted electrons are here indirectly enclosed in the pre-exponential factor and this allows to better explicit the couplings between discrete quantum states which are more effective for the electronic rearrangements within the collision complex^15^.

The important new aspect, already highlighted in a previous work where we discussed atom–atom systems^15^, is that here we are able to evaluate the relative role of two mechanisms for each state-to-state channel with its dependence on the collision energy *E*, or on the distance range of *R* mainly probed, and on the molecular orientation defined by *θ.*

**Figure 2:**
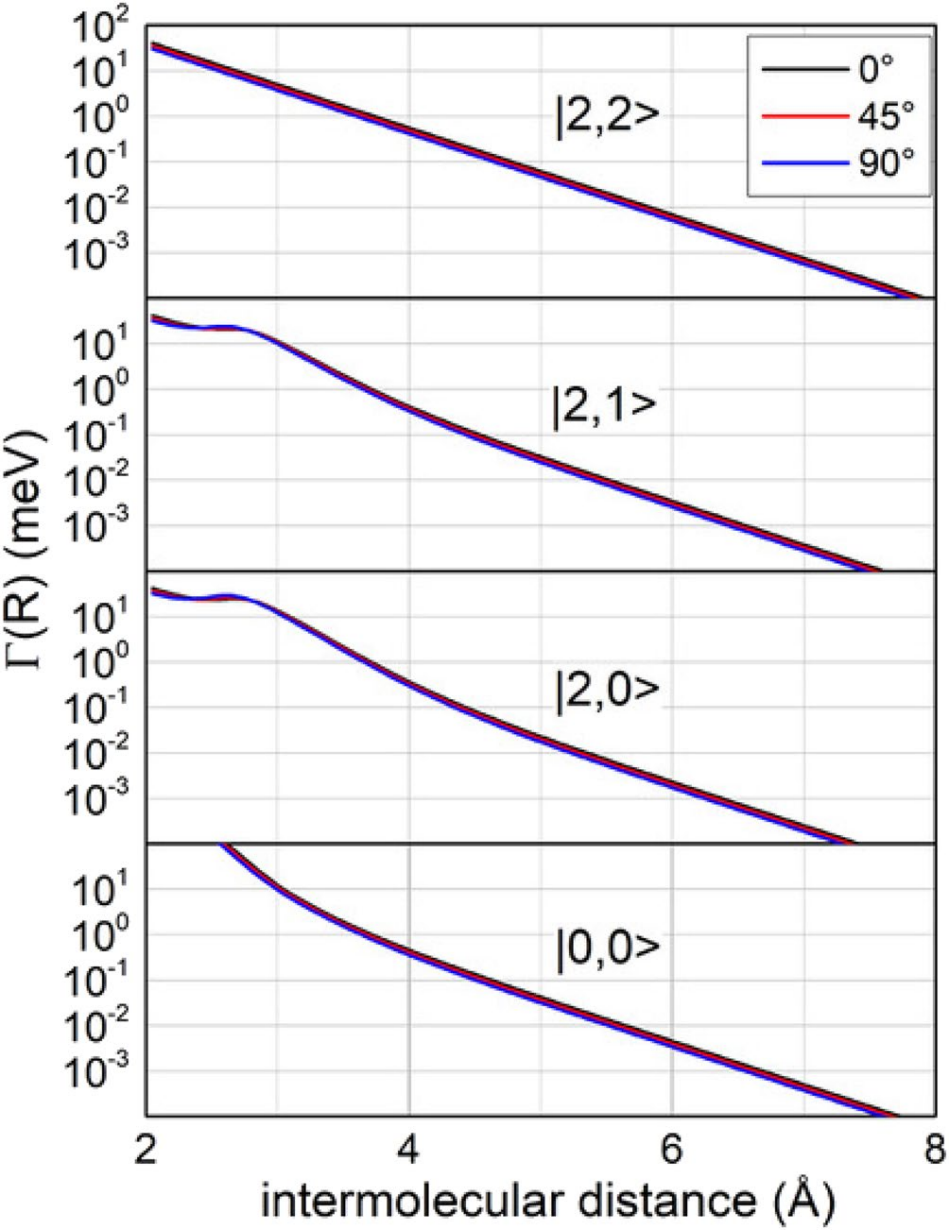
State-to-state *Γ* components obtained for different molecular orientation and plotted as a function of the intermolecular distance *R.* The quantum state $|J,\varOmega >$ of Ne^*^ reagent is indicated in the Figure while the ionic product N_2_^+^ is formed in the ground electronic state ^2^Σ_g_^+^. Note also that, because of the symmetry of atomic and molecular orbital involved in the precursor state, $|2,2 >$ reacts exclusively by an indirect mechanism (see text).

State-to-state *Γ* components, obtained applying Eq. (2), are plotted in Fig. 2. From such Figure some peculiar features merit to be emphasized:

1. Because of its nature controlled by physical interaction forces, $\varGamma_{|2,2 \to ions > }$ is the component more effective at long range;
2. Instead, $\varGamma_{|0,0 \to ions > }$ dominates at short separation distance, where the *marker-tracing* coefficient $C_{x}$ becomes 1 being more effectively determined by chemical forces;
3. $$\varGamma_{|2,0 \to ions > }$$ and $$\varGamma_{|2,1 \to ions > }$$ exhibit an intermediate behavior, modulated by the $$C_{x}$$ change with *R*, taking into account that at short *R* they become pure states of П symmetry ($$C_{x} = 0)$$;
4. Because of the symmetry of the molecular orbital which allows the electron removing under several configurations, including parallel and perpendicular geometries^27^, the present imaginary components exhibit dependence on the molecular orientation rather smaller respect to that found for polar hydrogenated molecules as water and ammonia^26^;
5. The state average value of *Γ* is in the right scale of that empirically obtained in ref. 21;
6. Finally, although *Γ* shows a limited dependence on *θ*, its role is strongly affected by the pronounced angular dependence of $V_{t} \left( {R,\theta } \right)$ which, at the same value of *E*, modulates the *R* ranges where the reaction is triggered.

## Results and discussion

Real and imaginary parts of the optical potential *W* are formulated (see SI for all details) according to a semi-empirical (phenomenological) method exploiting fundamental physical properties of involved partners^15,16,28^. A combined analysis of several experimental observables, probing simultaneously different region of *W*, is adopted to test its formulation and to fine tuning involved potential parameters. Cross sections are calculated within a semi-classical treatment of scattering events^7^. Such treatment, correctly operating for *E* ranging from sub-thermal (few meV) up to hyper-thermal (some eV) values, is proper to study the phenomena mentioned above, except those occurring under ultra-cold conditions, for which a full quantum approach is necessary.

**Figure 3:**
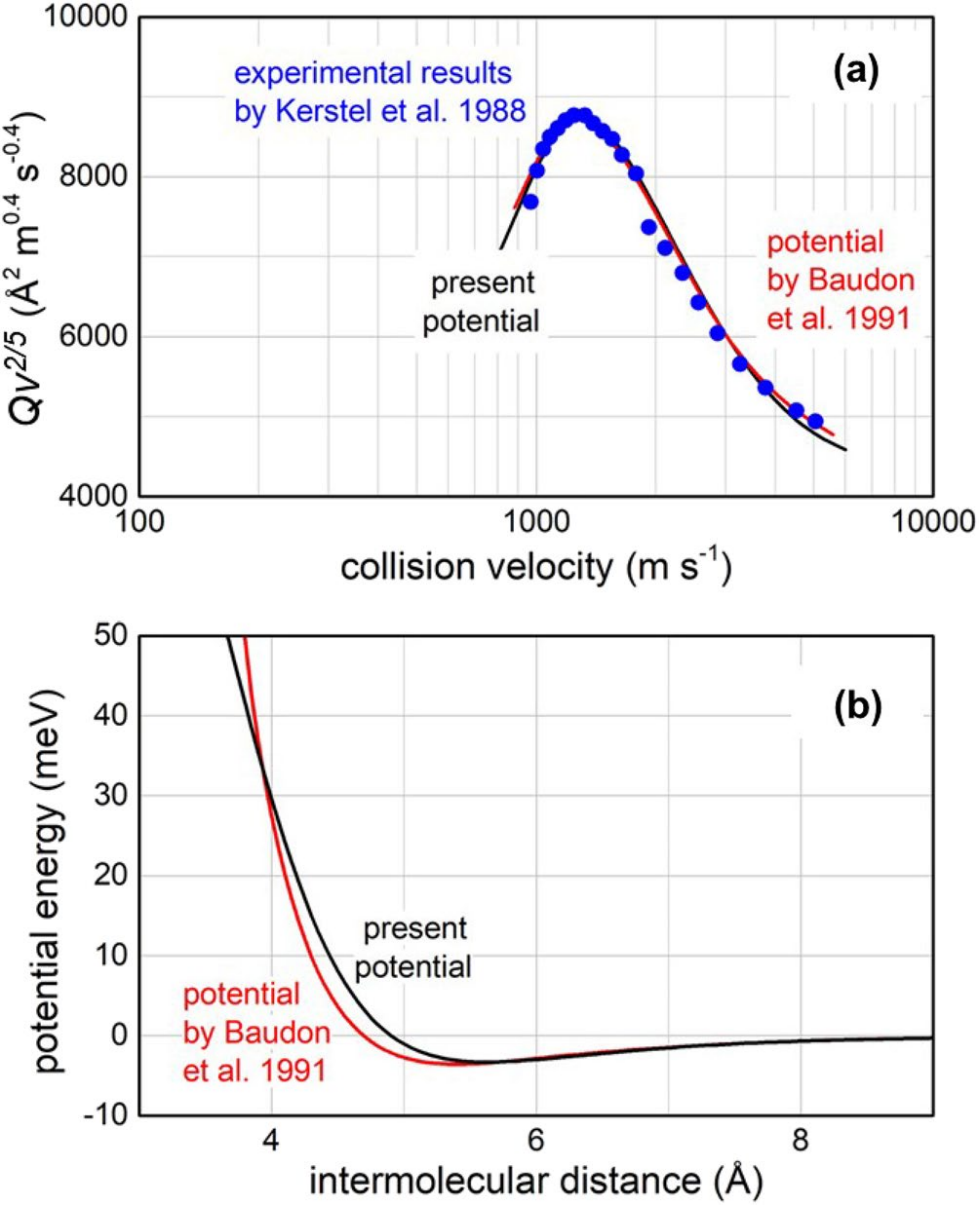
(**a**) Integral cross sections *Q*, measured as a function of the collision velocity *v*^19^, plotted as *Qv*^*2/5*^ to emphasize the “glory” interference pattern, compared with calculations, carried out within the semi-classical JWKB approximation^21^, by the present optical potentials for Ne^*^ (^3^P_2,0_) atoms scattered by N_2_. (**b**) The long-range behavior of isotropic (spherically averaged) real part of the optical potential in the entrance channels, compared with that of ref.^21^.

The Fig. 3a reports the total (elastic + inelastic) integral scattering cross section *Q*, measured as a function of the collision velocity *v*, with the resolution of the “glory” quantum interference effect^20^. Such observable directly probes the long-range attraction strength in entrance channels and the averaged potential well located at *R* ≃ 5.5 Å, where anisotropic interaction effects are vanishing. The Fig. 3b compares the present isotropic (averaged) potential with that of a previous analysis^21^: the agreement extended also to the first repulsive region indicates that also the present potential is expected to be consistent with total differential elastic cross sections measured at two different *E* values (71 and 295 meV)^21^.

**Figure 4:**
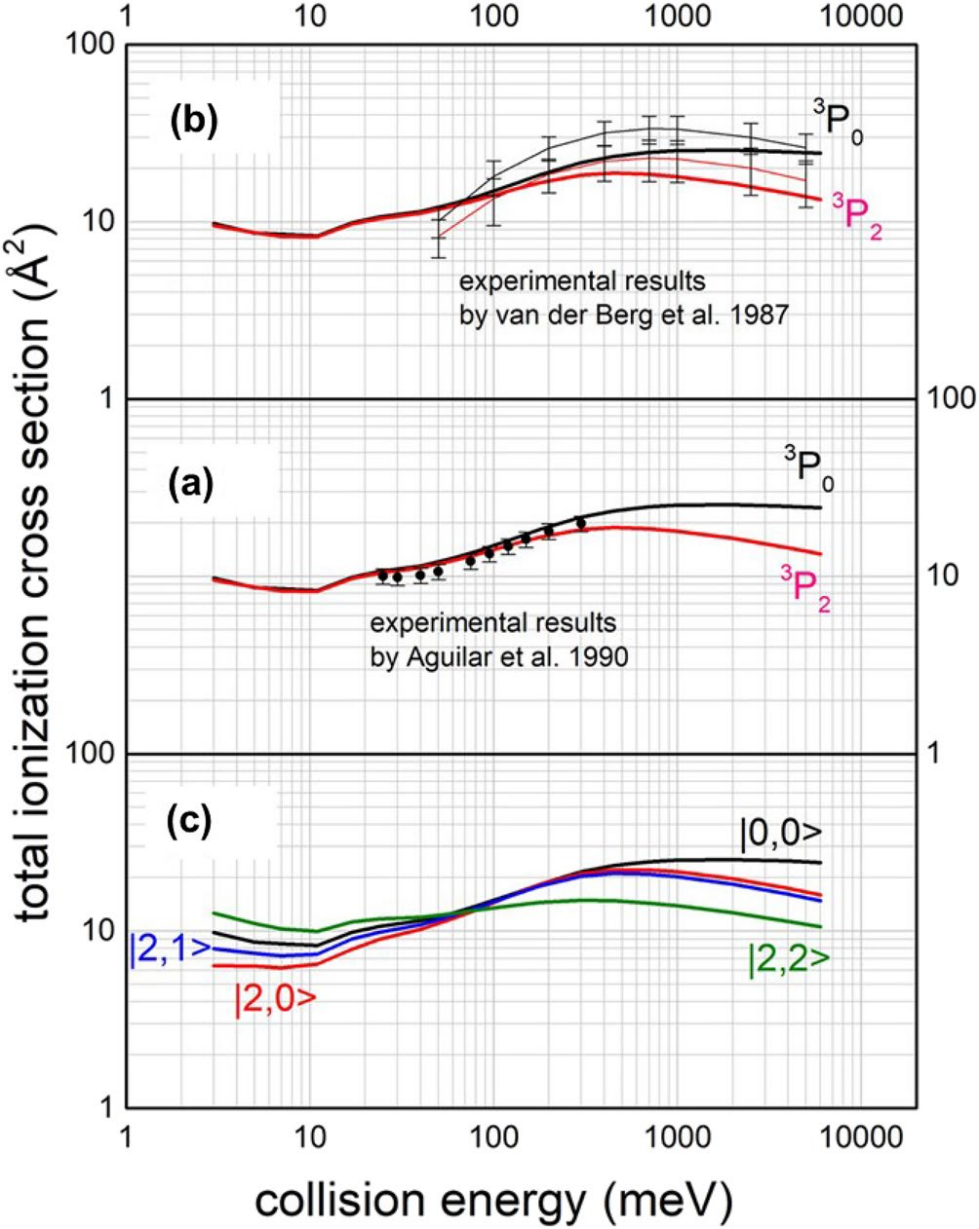
Total ionization cross sections for Ne^*^(^3^P_2,0_)-N_2_ as a function of the collision energy. (**a**) The full circles are results from the Perugia laboratory^29^. Marked red and black lines indicate the calculations of the present study carried out within the semiclassical approach^6,7^ and according to Eq. (3). (**b**) thin-pink and thin-black lines represent polynomial functions interpolating data measured in the Eindhoven laboratory^18^. Vertical error bars are representative of the uncertainties on the absolute scale of cross sections. Full lines are the same of (**a**) panel. (**c**) the contribution from each entrance channel leading to the formation of N_2_^+^ in its ground ^2^Σ_g_^+^ electronic state.

Total ionization cross sections *σ* have been measured, as a function of *E*, in our laboratory^2^ in the thermal range (see Fig. 4a) and in the Eindhoven laboratory^18^ from thermal up to the hyper-thermal conditions (see Fig. 4b). Note that while the energy dependence of *σ* is affected, in the probed range of *R*, by strength and anisotropy of ${V}_{t}$, its absolute value is directly controlled by the *Γ* behaviour. Respect to *Q*, *σ* more effectively depends on the interaction components emerging at shorter *R*, where their anisotropic behaviour is prominent. Moreover, ionization is a fast process occurring in a time scale of 10^−14^–10^−15^ s: it depends on $\frac{\hbar }{\varGamma }$ ratio and is shorter of typical molecular rotation periods (comparable or longer than 10^−12^ s). Such process is also triggered in restricted *R* ranges, confined in proximity of trajectories turning points which depend on *E* and on the orbital angular momentum of the collision complex^6,7^. Usually, ionization involves fixed molecular orientations.

The collision dynamics regime, accounting for all boundaries specified above, describes *σ* as a proper average over contributions from all molecular orientations effectively assumed by N_2_ molecule within the precursor state. Therefore, *σ* is defined according to a “piloted” Infinite Order Sudden Approximation (IOSA) as:

3 $$ \sigma \left( E \right) = \left[ {\frac{1}{{\cos \left( {0^{^\circ } } \right) - \cos \left( {45^{^\circ } } \right)}}\mathop \smallint \limits_{{0^{^\circ } }}^{{45^{^\circ } }} \sigma \left( E \right)sin\left( \theta \right)d\theta } \right]f_{0} \left( E \right) + \left[ {\mathop \smallint \limits_{{0^{^\circ } }}^{{90^{^\circ } }} \sigma \left( E \right)sin\left( \theta \right)d\theta } \right]\left( {1 - f_{0} \left( E \right)} \right) $$

Note that $f_{0} \left( E \right)$ = $\frac{1}{{1 + e^{{\frac{{E - E_{0} }}{Et}}} }}$ is a Fermi weight function where the values of *E*_*0*_ and *E*_*t*_ factors, defining, respectively the value of *E* where the two integrals exhibit the same weight and how fast the passage between the two calculation methods occurs, depending on the interaction anisotropy and on the average rotation energy of N_2_. In the present study and ${E}_{0}$ and ${E}_{t}$ have been fixed at 80 and 60 meV, respectively.

The first integral in Eq. (3) describes the average over the orientations, preferentially assumed by the molecule in the precursor state formed at low *E* (for *E* < *E*_*0*_), where molecular alignment effects within the intermolecular electric field gradient are more effective^30,31^. The second integral, prevalent at high *E* (for *E* > *E*_*0*_), represents the “canonical” formulation of IOSA, where all molecular orientations contribute to the process, according to their random-statistical weights. The good description of the experimental observables provided by the present analysis is shown in the Fig. 4. Note that for *E* > 100 meV, the formation of N_2_^+^ product in the excited electronic state (A^2^П) becomes also permitted, although it is expected to provide minor contributions. Moreover, in a wide range of *E* a simplified version of “piloted” IOSA treatment provides results coincident with those of Eq. (3). In this simpler treatment, *σ* is defined as the weighted averaged over the contributions from a limited number of selected angles *θ*, assumed by the molecule within the precursor state (see below for its utility). Accordingly, *σ* at each *E* is provided by:

4 $$ \sigma \left( E \right) \cong \frac{1}{9} \sigma_{{0^{^\circ } }} + \frac{2}{9} \sigma_{{25^{^\circ } }} + \frac{2}{9} \sigma_{{45^{^\circ } }} + \frac{2}{9} \sigma_{{60^{^\circ } }} + \frac{2}{9} \sigma_{{90^{^\circ } }} $$

### Selectivity of the collision dynamics

 The present treatment gives a proper rationalization of the higher reactivity of Ne^*^(^3^P_0_) with respect to Ne^*^(^3^P_2_) reagent, emerging for *E* higher than 100 meV (see Fig. 4a,b). In particular, this experimental finding arises from the raising of $$C_{x}$$ coefficient, as the probed *R* range decreases, and this determines an increasing role of *A*_*DM*_ coupling just for the $$|0,0 \to ions >$$ channel (see Eq. (2)). The origin of this experimental finding emerges also in Fig. 4c where the dependence of the reactivity on the $$|J,\varOmega >$$ atomic sublevels of Ne^*^reagent is reported as a function of *E*. As for atom–atom CHEMI, $$ |0,0 >$$ is the entrance atomic state that at short *R* correlates with a molecular state of Σ symmetry^15,16^, where increasing alignment of the half-filled *p* orbital of the open shell Ne^+^ along **R** stimulates *DM* by CT effects. By contrast, $$|2,2 >$$ shows a completely different collision energy dependence of *σ* since it is the atomic state of Ne^*^ reagent (correlating with that of Ne^+^) for which the half-filled *p* orbital maintains an alignment perpendicular to **R,** determining the formation of a pure molecular state of Π symmetry^15^. Therefore, it can react at any *E* exclusively through *IM*.

**Figure 5:**
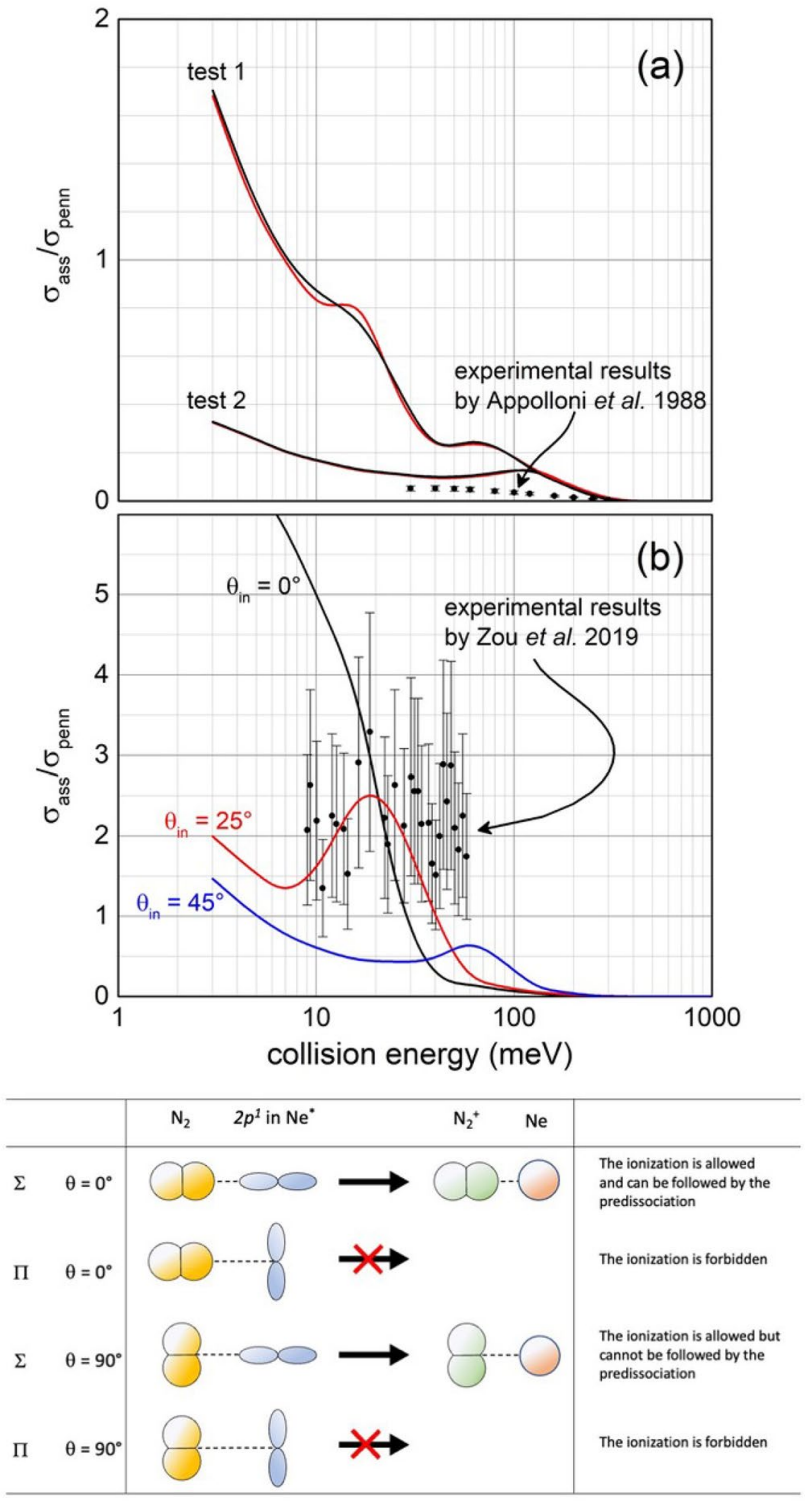
(**a**) Comparison of predicted associate/penning branching ratio (see text) with experimental data from Perugia laboratory^32^. (**b**) Comparison of an exclusive *concerted flip* mechanism (see text) with data from Lausanne laboratory corrected for predissociation^24^. In such panel, the role of the initial molecular orientation is emphasized; as in Fig. 4, marked red and black lines indicate the calculations performed in the case of collisions involving the ^3^P_2_ and ^3^P_0_ spin–orbit states of Ne^*^, respectively. *Lower panel*: A schematic view of precursor state structures promoting reaction and associated ion formation. Note that while the half-filled *p* orbital of Ne^*^ atom exhibits a nodal plane, the HOMO orbital of N_2_ from which the electron is removed, does not exhibit any nodal plane perpendicular to the bond axis and passing for its center of mass^33^. Therefore, in the second and fourth cases, characterized by overlap null between orbitals exchanging the electron, the ionization by direct mechanism is forbidden and the reaction can occur only by indirect mechanism (see text).

Other selectivity emerge from the analysis of the *associative to penning* $\left( {\frac{{\sigma_{ass} }}{{\sigma_{pe} }}} \right)$ ratio. The Fig. 5a compares results predicted by Eq. (4) (test 1) with experimental data measured in our laboratory with effusive MB^32^. The pronounced difference between predictions and observables suggests that a consistent fraction of associated NeN_2_^+^ ions undergo predissociation as a consequence of the vibrational/rotational excitation of the formed N_2_^+^ moiety within (NeN_2_)^+^. As for many other inelastic events, the possibility of internal degree excitation of nascent N_2_^+^ (see Refs.^19,22^), strongly depending on geometry of the precursor state assumed during ionization, is favoured for collinear or near collinear configurations. Also, for the present CHEMI an improved agreement with the experimental data is obtained when assuming that only approaches with θ = 60° and 90° are effective for the formation of the associated ion without predissociation (see test 2 in Fig. 5a). Under such conditions predicted results are also in the right scale of quantum state averaged data from Ref.^23^.

The important role of the predissociation has been recently emphasized in an interesting investigation of the Lausanne group^24^, carried out with state selection of Ne^*^(^3^P_2_) reagent and with N_2_ relaxed in low lying rotational states, populated after supersonic expansion leading to the formation of seeded MB. An intriguing finding emerges from the comparison of the highest measured $$\frac{{\sigma }_{ass}}{{\sigma }_{pe}}$$ ratio, referred to the $$ |2,2 >$$ entrance channel and corrected for predissociation (see Fig. 5b), with the results of our analysis. As stressed before, the $$|2,2 >$$ state involves the half-filled *p* atomic orbital of Ne^*^ reagent aligned perpendicularly to **R.** Moreover, under thermal and sub-thermal conditions of *E,* probed in Lausanne experiments, the intermolecular electric field gradient tend to efficiently align the rotationally cold N_2_ molecules flying in the used seeded MB (the estimated final rotation temperature is ~ 20 K). Such conditions favour the formation of a precursor state with initial collinear or near collinear configurations that are the most effective for ro-vibration excitation. Under such conditions $$ A_{DM}$$ is zero because the absence of the overlap between orbitals exchanging the electron. The reaction is then exclusively promoted by *A*_*IM*_ (see Eq. (2)), usually determined by electronic cloud polarization, perturbation-change in angular momentum couplings, and Coriolis effects^14–16^. Therefore, the reactivity in the $$|2,2 >$$ entrance channel, hindered for *DM*, can be triggered by an *exclusive flip* of the atomic orbital alignment and/or by a less probable *concerted flip* of atomic and molecular alignment. In the present case we tentatively attribute the origin of these *flips* mostly to Coriolis effects, stimulated both by centrifugal effects of orbital angular momentum of the collision complex and by rotational/librational motions of N_2_ trapped within the precursor state. Moreover, while *exclusive flip* indirectly promotes reaction with pronounced predissociation, because of the combined excitation of the internal degree of freedom of products, the *concerted flip* leads to stable associated ion in the perpendicular configuration (see lower panel of Fig. 5). Assuming that reaction occurs exclusively via a *concerted flip*, predicted ratios (see Fig. 5b, where the role of the precursor state geometry is emphasized) appear in better agreement with data, corrected for predissociation^24^.

## Conclusions

Extending and generalizing the treatment recently proposed for atom–atom reactions^15,16^, an internally consistent formulation of real and imaginary parts of the anisotropic optical potential is obtained for a prototype atom-molecule CHEMI. The proposed formulation allows to rationalize for the first time simultaneously several experimental findings obtained in our and other laboratories with the MB technique. Accordingly, the leading interaction components, stimulating the electronic rearrangements within the precursor state, have been properly characterized and an important *marker-tracing* coefficient has been isolated that allows to defines at each collision energy *E* the type of electronic rearrangement dominant and the relative role of direct and indirect mechanism along each state-to-state reaction channel.

In conclusion, this new methodology, for the first time applied to the simple Ne^*^-N_2_ system, provides unique information on the stereo-dynamics of state-to-state CHEMI reactions as prototype gas-phase oxidation processes involving molecules. Obtained results are fundamental to cast light on the stereodynamics of other chemical reactions^1,34^ for which structure and stability of the precursor state, controlled by alignment/orientation of reagents, and electronic rearrangements in TS, with changes in angular momentum couplings, are more difficult to isolate.

## Supplementary Information

Supplementary Information.
