# Supplementary material for: Selectivity of weak intermolecular forces and precursor state of elementary oxidation reactions, a new insight on Ne* + N2 chemiionization
Source: Sci Rep. 2021 Sep 27;11:19105. doi: 10.1038/s41598-021-98602-8 (PMC8476572; doi:10.1038/s41598-021-98602-8)
Supplement: Supplementary file 1 — Supplementary Information. [file 41598_2021_98602_MOESM1_ESM.docx]

**Supplementary Information** to the paper:

**Selectivity of weak intermolecular forces and precursor state of elementary oxidation reactions, a new insight on Ne^*^+N_2_ chemiionization**

*Stefano Falcinelli,^1,*^ Franco Vecchiocattivi,^1^ and Fernando Pirani^1,2^*

**The interaction potential in entrance and exit channels**

The adopted formulation of the real component $V_{t}$of the optical potential, providing in the entrance channels its dependence on the intermolecular distance *R* and on the angle *θ*, defining the molecular axis (bond) orientation respect to **R**, exploits this expression [1,2]:

$$V_{t}\left( R,\theta\right)= {S\left( R \right) V(R,\theta)}^{neut.-neut.}+\left( 1-S\left( R \right) \right) V{(R,\theta)}^{ion-neut.}$$

where ${V(R,\theta)}^{neut.-neut.}$and $V{(R,\theta)}^{ion-neut.}$ have been represented by the Improved Lennard Jones function (ILJ). Its general form is [3,4]:

$$V_{ILJ}\left( R,\theta\right)=\varepsilon\left( \theta\right)\left[ \frac{m}{n\left( R,\theta\right)-m}\left( \frac{R_{m}\left( \theta\right)}{R} \right)^{n\left( R,\theta\right)}-\frac{n\left( R,\theta\right)}{n\left( R,\theta\right)-m}\left( \frac{R_{m}\left( \theta\right)}{R} \right)^{m} \right]$$

with

*n*$\left( R,\theta\right)= \beta+4\left( \frac{R}{R_{m}\left( \theta\right)} \right)^{2}$

The basic parameters $ɛ\left( \theta\right)$ and $R_{m}\left( \theta\right)$, representing the potential well depth and its location at each *θ,* are here represented by an expansion in Legendre polynomials:

$R_{m}\left( \theta\right)$= $R_{m}$ +$R_{m_{a}}\cdot P_{2}\left( \cos\theta\right)$ ; $ɛ\left( \theta\right)=ɛ+ ɛ_{a}\cdot P_{2}\left( \cos\theta\right)$

As usual, *n*$\left( R,\theta\right)$ defines hardness of the repulsive wall and radial and angular modulation of the attraction. As previously [1], the switching function *S*$\left( R \right)$, that accounts for the transition from the neutral-neutral to the ion-neutral representation of the interaction, has been defined as:

$$S\left( R \right) =\frac{1}{1+ e^{\left( \frac{R_{o}-R}{d} \right)}}$$

While $R_{o}$is the distance where the two combined limiting potential forms have the same weight, *d* describes how fast the transition occurs.

However, for the complete representation of $V_{t}$it is useful to take into account additional aspects:

1. At large *R*, where the neutral-neutral representation is dominant and the system exhibits a substantial behavior typical of an alkaline atom interacting with a diatomic partner, the ILJ formulation of the interaction with m = 6 appears to be complete.
2. At intermediate and short *R*, in addition of the molecular anisotropy, the emerging contributions of the open shell “P” metastable atom [5], due to anisotropic role of its ionic core, must be properly taken into account.
3. In the exit channels the outgoing anisotropic interaction $V_{out}$ is confined in that of an open shell molecular ion-noble gas atom system, for which ILJ assumes m = 4.

Therefore, generalizing the atom-atom approach and following the guidelines extensively developed in our laboratory [5-7], it is possible to describe the interaction energy, when an open “P” shell atom or ion approaches a closed shell molecular species, by effective adiabatic potential energy surfaces (PES), that include the contributions associated to the different symmetries of the molecular states of the tri-atomic complex, mixed by spin-orbit couplings.

In the development of the interaction potential representation, it is convenient to assume *in the* *first step* N_2_ and N_2_^+^ behaving as isotropic partners and *in the second step* to introduce the role of molecular orientation. Therefore, *in the first step* the adopted formulation, depending only on *R*, must be the same adopted for atom-atom systems [8]. Accordingly, the PES degenerate in a manifold of potential curves. They can be then formulated in terms of Σ and Π molecular states, defined by the electronic quantum number Λ=0 and Λ=1 and identified as $V_{\Sigma}$and $V_{\Pi}$ interaction components, mixed by spin-orbit effects [5,9]. It is proper to employ for such a description a weighted sum of $V_{0}$and $V_{2}$Legendre-expansion radial coefficients, defined as $V_{0}=\frac{1}{3}\left( V_{\Sigma}+2V_{\Pi} \right)$ and $V_{2}=\frac{5}{3}\left( V_{\Sigma}-V_{\Pi} \right)$. The inverse formulas are simply given by $V_{\Sigma}=V_{0}+ \frac{2}{5}V_{2}$and $V_{\Pi}=V_{0}- \frac{1}{5}V_{2}$. In this way, $V_{0}$describes the interaction component, spherically averaged over all quantized orientations permitted to the half-filled 2p orbital of open shell Ne^*^ reagent, within the complex formed with an isotropic partner. Moreover, all anisotropic contributions, arising from quantized Σ and Π symmetries, are directly taken into account through the use of the anisotropic term$V_{2}$. In particular, the configuration interaction, that couples by charge transfer (CT) Ne^+^-N_2_ and Ne-N_2_^+^ states, affects more effectively the state of Σ symmetry, since it is that exposing the half-filled orbital aligned along **R**. In the case of ^3^P_J_ (or ^2^P_J_) open-shell atomic species, like Ne^*^ (or Ne^+^), characterized by a reversed sequence of spin-orbit sublevels, the effective adiabatic potential energy curves $V_{|J,\Omega˃}$, that represent the radial components of the manifold of the PES, obtained averaging over the molecular orientations, are defined for all channels [8,9] as:

$$V_{|0,0>} = V_{0}+ \frac{1}{10} V_{2}+ \frac{1}{2}\Delta_{0}+ \frac{1}{2}\left( \frac{9}{25} V_{2}^{2} +\Delta_{0}^{2} - \frac{2}{5}V_{2}\Delta_{0} \right)^{1/2}$$

$$V_{|2,0>} = V_{0}+ \frac{1}{10} V_{2}+ \frac{1}{2}\Delta_{0}- \frac{1}{2}\left( \frac{9}{25} V_{2}^{2} +\Delta_{0}^{2} - \frac{2}{5}V_{2}\Delta_{0} \right)^{1/2}$$

$$V_{|2,1>} = V_{0}+ \frac{1}{10} V_{2}+ \frac{1}{2}\Delta_{1} - \frac{1}{2}\left( \frac{9}{25} V_{2}^{2} +\Delta_{1}^{2} \right)^{1/2}$$

$$V_{|2,2>} = V_{0}- \frac{1}{5} V_{2}$$

where Δ_0,_ ∆_1_ are the energy splitting between fine atomic sublevels of Ne^*^(^3^P_J_) reagent [8].

As indicated above, in the entrance channels the $V_{0}$term, coinciding with $V_{t}\left( R \right)$, exhibits a mixed nature, accounting for the gradual passage from neutral-neutral to ion-neutral system (where the outer electron *3s* is strongly polarized), as *R* decreases [1]. Details on the correlation diagram between neutral-neutral and ion-neutral states are given in ref. 8.

In the exit channels, a typical isotropic molecular ion-closed shell atom operates that is stabilized by the configuration interaction by CT. Therefore, $V_{out}$for outgoing is represented as:

$$V_{out}=V_{0_{out}}-\frac{2}{5}V_{2}$$

As stressed above, both $V_{0}$ and $V_{0_{out}}$ terms have been defined as combination or simple ILJ functions, respectively. Moreover, for both entrance and exit channels, $V_{2}$, that accounts for all anisotropic interaction contributions due to configuration interaction, has been represented by an exponential decreasing function, defined by a pre-exponential factor *A* and an exponent *α,* plus an additional contribution $\frac{C_{a}}{R^{6}}$[8]. For entrance and exit channels the modulus of the exponential function is the same, while its main role on the interaction is negative for exit and positive for entrance, since related to *bonding* and *antibonding* stabilization effects by CT that arise from the configuration interaction between entrance and exit channels of the same symmetry [5,8,9]. The additional contribution accounts for the role of polarizability anisotropy of Ne^*^(^3^P) on asymptotic behavior of$V_{2}$*.*

Moreover, for entrance channels involving the passage from neutral-neutral and ion-neutral representation [8], it has been also found that the Σ and Π character of the permitted quantum states can be defined, respectively, by the marker coefficients $C_{x}$ and $\left( 1-C_{x} \right)$. For involved potential energy curves $V_{|J,\Omega˃}$ at all *R* values such coefficients are evaluated exploiting the following relations [6]:

$${{V_{|2,0>},V}_{|2,1>} ,V}_{|3/2,1/2>} = {cos}^{2}\alpha V_{\Sigma}+ {sin}^{2}\alpha V_{\Pi}$$

$${V_{|0,0>},V}_{|1/2,1/2>} = {sin}^{2}\alpha V_{\Sigma}+ {cos}^{2}\alpha V_{\Pi}$$

Where:

$$\cos^{2}\alpha= \frac{1}{2} +\frac{\left( 1-\frac{9V_{2}}{5\Delta} \right)}{4\sqrt{2} \sqrt{1+\left[ \left( \frac{1-\frac{9V_{2}}{5\Delta}}{2\sqrt{2}} \right) \right]^{2}}}$$

with $V_{2}$ here including only the exponential component due to the configuration interaction.

These formulas agree with the following asymptotic conditions: at short distances, all potential energy curves must represent states having a pure Σ or Π character, while at large distances, where the spin-orbit coupling is dominant, a mixing of the characters occurs. The behavior of $V_{|2,2>}$curve, correlating at short *R* with $V_{|\frac{3}{2},\frac{3}{2}>}$ curve, is not discussed in detail because it shows at all distances a pure Π character. Additional details on the adiabatic correlation between atomic and molecular states, defined in terms of proper quantum number, have been extensively discussed in recent papers [8,10,11], where also the meaning of *C_x_* and (1-*C_x_*) coefficients, with their relevance in the formulation of state-to-state *Γ* (see the main text) is justified. Such coefficients must be considered as the proper markers of the system evolution along each reaction channel.

It has been also demonstrated [5,7,9] that *non adiabatic* effects, promoted by changes in the electronic angular coupling schemes, describing the transition from atom-atom to molecular states, manifest with the highest probability at a distance where $\left| V_{2} \right|$is comparable with $\Delta_{i}.$

*In the second step*, the extension of the formulation, in order to account for anisotropic behavior of N_2_ and N_2_^+^ within the interacting complex, depending on the orientation angle *θ,* is simply obtained expanding the interaction components in terms of Legendre polynomials. For $V_{0}\left( R,\theta\right)$ = $V_{t}\left( R,\theta\right)$ the formulation has been discussed in detail above, to which must be added in the specific case of Ne^+^-N_2_, the strongly anisotropic electrostatic interaction, $V_{electr},$ between the charge *q* on emerging Ne^+^ ion and the permanent electric quadrupole *Q* on N_2_, defined as

$V_{elect}$ $\left( R, \theta\right)=-\frac{q\cdot Q}{R^{3}}P_{2}\left( \cos\theta\right)$

We assigned to the emerging Ne^+^, in the strongly polarized Ne^*^atom, an effective electronic polarizability of about 2.7 au and a value of *q*=0.9 au (see also ref. [1]), while the molecular *Q* has been fixed to the value of 1.1 au [11].

For $V_{0_{out}}\left( R,\theta\right)$ an anisotropic ILJ function has been used, where again:

$R_{m}\left( \theta\right)$= $R_{m}$ +$R_{m_{a}}\cdot P_{2}\left( \cos\theta\right)$ ; $ɛ\left( \theta\right)=ɛ+ ɛ_{a}\cdot P_{2}\left( \cos\theta\right)$

Finally, for $V_{2}\left( R,\theta\right)$ we adopt

$$V_{2}\left( R,\theta\right)=V_{2}\left( R \right)+ V_{2_{a}}\cdot P_{2}\left( \cos\left( \theta\right) \right)+\frac{c_{a}}{R^{6}}$$

where $V_{2_{a}}$ exclusively affects the angular dependence of the pre-exponential factor *A*.

The values of the potential parameters are given in Table S1. They have been obtained semi-empirically following the guidelines described in refs [5-9,11] that have been suggested by the phenomenological investigation of several systems involving open-shell atoms, ions and diatomic partners.

**The state-to-state** $\boldsymbol{\Gamma}_{\boldsymbol{|J,\Omega\to ions˃}}$ **components**

The main text of the paper reports both the plot of the real $V_{t}\left( R,\theta\right)$ part of the optical potential, including also the interaction in the exit channel, and the formulation of the imaginary part *Γ*$\left( R,\theta\right)$provided as combination of entrance-exit channels coupling terms, $A_{DM}$and $A_{IM},$ weighted on the marker coefficients $C_{x}$ and $\left( 1-C_{x} \right)$.

Obtained $\Gamma_{|J,\Omega\to ions˃}$ components are plotted in Figure 2 of the main text.

The important new aspect is that here we are able to evaluate the relative role of two mechanisms for each state-to-state channel with its dependence on the collision energy, or on the distance range of *R* mainly probed, and on the molecular orientation defined by *θ.* In particular, simple-operative exponential functions have been adopted for $A_{DM}$ and $A_{IM}$ coupling terms. The first term represents a defined fraction of $V_{2}$ exponential component, while the second has been obtained generalizing criteria given in refs. [8-10]. The coupling terms $A_{DM}$and $A_{IM}$ have been represented by exponential functions with values of pre-exponent factor *A* and of exponent *α* given in Table S1. It is important to remark again that overlap effects with the continuum wave function of emitted electrons are here indirectly enclosed in the pre-exponential factor and this allows to better explicit the couplings between discrete quantum states which are more effective for the electronic rearrangements within the collision complex.

TABLE S1: Parameters of the optical potentials.

| **Incoming potential (Ne^*^-N_2_)** | | | | | | | |
| --- | --- | --- | --- | --- | --- | --- | --- |
| *neutral-neutral component* | | |  | | *ion-neutral component* | | |
| $R_{m}$(Å) | 5.70 | |  | | $R_{m}$(Å) | | 2.96 |
| $R_{m_{a}}$(Å) | 0.00 | |  | | $R_{m_{a}}$(Å) | | 0.35 |
| $\varepsilon$(meV) | 3.15 | |  | | $\varepsilon$(meV) | | 114.0 |
| $\varepsilon_{a}$(meV) | 0.00 | |  | | $\varepsilon_{a}$(meV) | | 16.0 |
| $\beta$ | 7.00 | |  | | $\beta$ | | 8.00 |
| *m* | 6.00 | |  | | *m* | | 4.00 |
| **Switching function** | | | | | | | |
| $R_{0}$(Å) | | 3.72 | |  |  |  |  |
| *d*(Å) | | 0.50 | |  |  |  |  |
|  | |  | |  |  |  |  |
| **Electronic anisotropy** | | | |  |  |  |  |
| $V_{2}$(meV) | | 1.40$\times$10^7^ | |  |  |  |  |
| $V_{2_{a}}$(meV) | | 4.00$\times$10^6^ | |  |  |  |  |
| $\alpha$(Å^-1^) | | 4.32 | |  |  |  |  |
| *c_a_*(meV Å^-6^) | | 1.30$\times$10^3^ | |  |  |  |  |
|  | |  | |  |  |  |  |
| **Outgoing potential (Ne-N_2_^+^)** | | | |  |  |  |  |
| $R_{m}$(Å) | | 3.30 | |  |  |  |  |
| $R_{m_{a}}$(Å) | | 0.35 | |  |  |  |  |
| $\varepsilon$(meV) | | 30.0 | |  |  |  |  |
| $\varepsilon_{a}$(meV) | | 13.2 | |  |  |  |  |
| $\beta$ | | 8.50 | |  |  |  |  |
| *m* | | 4.00 | |  |  |  |  |
|  | |  | |  |  |  |  |
| **Entrance-exit channels couplings** | | | |  |  |  |  |
| *A_DM_*(meV) | | 5.00$\times$10^6^ | |  |  |  |  |
| *A_DMa_*(meV) | | 5.00$\times$10^5^ | |  |  |  |  |
| $\alpha_{DM}$(Å^-1^) | | 4.32 | |  |  |  |  |
| *A_IM_*(meV) | | 1.90$\times$10^3^ | |  |  |  |  |
| *A_IMa_*(meV) | | 3.80$\times$10^2^ | |  |  |  |  |
| $\alpha_{IM}$(Å^-1^) | | 2.20 | |  |  |  |  |

**References**

[1] B. G. Brunetti et al. The Stereodynamics of Penning ionization of water by metastable neon atoms. *J. Chem. Phys.* **139**, 164305 (2013).

[2] S. Falcinelli, et.al. The electron couplings in the transition states: the stereodynamics of state to state autoionization processes. *J. Chem.* *Phys.***150,** 044305 (2019).

[3] F. Pirani et al. An Atom-bond pairwise additive representation for intermolecular potential energy surfaces. Chem. Phys. Lett. **394**, 37-44 (2004).

[4] F. Pirani et al. Beyond the Lennard Jones model: a simple and accurate potential function probed by high resolution scattering data useful for molecular dynamics simulations. *Phys. Chem. Chem. Phys.***10**, 5489-5503 (2008).

[5] F. Pirani et al. Experimental benchmarks and phenomenology of interatomic forces: open-shell and electronic anisotropy effects. *Int. Rev. Phys. Chem*. **25**, 165-199 (2006).

[6] P. Tosi et al. The reaction of argon ions with hydrogen and deuterium molecules by crossed beams: Low energy resonances and role of vibronic levels of the intermediate complex. *J. Chem. Phys.* **99**, 985-1003 (1993).

[7] F. Pirani et al. Coupling by charge transfer: role in bond stabilization for open-shell systems and ionic molecules and in harpooning and proton attachment processes. Mol. Phys. **98**, 1749-1762 (2000).

[8] S. Falcinelli et al. General treatment for stereodynamics of state-to state chemiionization reactions. Comm. Chem. **3,** 64 (2020).

[9] V. Aquilanti et al. Molecular beam studies of weak interactions for open-shell systems: The ground and lowest excited states of rare gas oxides. *J. Chem. Phys.* **89**, 6157-6164 (1988).

[10] S. Falcinelli et al. Adiabatic and Nonadiabatic Effects in the Transition States of State to State Autoionization Processes. *Phys. Rev. Lett.* **121**, 163403 (2018).

[11] S. Falcinelli et al. Quantum-State Controlled Reaction Channels in Chemi-ionization

Processes: Radiative (Optical−Physical) and Exchange (Oxidative−Chemical) Mechanisms. Acc. Chem. Res. **53,** 2248-2260 (2020)**.**
